# *Thymus satureioides* Coss.: Mineral Composition, Nutritional Value, Phytochemical Profiling, and Dermatological Properties

**DOI:** 10.3390/molecules28124636

**Published:** 2023-06-08

**Authors:** Ismail Mahdi, Nidal Fahsi, Hassan Annaz, Badreddine Drissi, Mustapha Barakate, Mona F. Mahmoud, Mansour Sobeh

**Affiliations:** 1 AgroBioSciences Department, College for Sustainable Agriculture and Environmental Science, Mohammed VI Polytechnic University, Ben Guerir 43150, Morocco; ismail.mahdi@um6p.ma (I.M.); mustapha.barakate@um6p.ma (M.B.); 2 Laboratory of Microbial Biotechnology, AgroSciences and Environment, CNRST Labeled Research Unit N°4, Faculty of Sciences Semlalia, Cadi Ayyad University, Marrakesh 40000, Morocco; 3 Department of Pharmacology and Toxicology, Faculty of Pharmacy, Zagazig University, Zagazig 44519, Egypt; mona_pharmacology@yahoo.com

**Keywords:** *Thymus satureioides*, dermatological, antibacterial activity, nutritional value, mineral content

## Abstract

Zaitra, *Thymus satureioides*, is an aromatic plant with a long history of use in traditional medicine. In this study, we assessed the mineral composition, nutritional value, phytocontents, and dermatological properties of the aerial parts of *T. satureioides.* The plant contained high contents of calcium and iron, moderate levels of magnesium, manganese, and zinc, and low contents of total nitrogen, total phosphorus, total potassium, and copper. It is rich in several amino acids, including asparagine, 4-hydroxyproline, isoleucine, and leucine, and the essential amino acids account for 60.8%. The extract contains considerable amounts of polyphenols and flavonoids (TPC = 118.17 mg GAE/g extract and TFC = 32.32 mg quercetin/g extract). It also comprises 46 secondary metabolites, identified through LC-MS/MS analysis, belonging to phenolic acids, chalcones, and flavonoids. The extract elicited pronounced antioxidant activities, inhibited the growth of *P. aeruginosa* (MIC = 50 mg/mL), and reduced biofilm formation by up to 35.13% using the ¼ sub-MIC of 12.5 mg/mL. Moreover, bacterial extracellular proteins and exopolysaccharides were diminished by 46.15% and 69.04%, respectively. Likewise, the swimming of the bacterium was impaired (56.94% decrease) in the presence of the extract. In silico, skin permeability and sensitization effects revealed that out of the 46 identified compounds, 33 were predicted to be exempt from any skin sensitivity risk (Human Sensitizer Score ≤ 0.5), while extensive skin permeabilities were observed (Log *K_p_* = −3.35–−11.98 cm/s). This study provides scientific evidence about the pronounced activities of *T. satureioides,* supports its traditional uses, and promotes its utilization in the development of new drugs, food supplements, and dermatological agents.

## 1. Introduction

*Thymus satureioides* Coss., commonly known as savory thyme, is a medicinal plant from the Lamiaceae family [1,2]. It is a perennial shrub that grows between 10 and 60 cm in height and is found exclusively in semiarid regions of the Moroccan High Atlas and Anti-Atlas. Locally, it is referred to as “Zaitra” or “Azkuni” [3,4,5]. Traditional uses of this plant include the treatment of various conditions such as diabetes, hypertension, fever, skin and blood circulation disorders, bronchitis, pain perception, immune system issues, pharyngitis, and influenza [6]. Furthermore, several studies have investigated the pharmacological properties of *T. satureioides* extracts and essential oils, revealing their potential as antidiabetic [2], anticancer [7], anti-inflammatory [8], antimicrobial [9,10,11], insecticidal [12,13], and hypolipidemic [14] agents. However, the underlying mechanisms responsible for most of these pharmacological effects have received limited research attention [6].

Like others, *T. satureioides* has gained attention for its dermatological applications due to its high content of antioxidants, antimicrobials, and anti-inflammatory compounds. Plant-based dermatological agents were shown to improve the overall health and appearance of the skin by providing nutrients, hydration, and protection from environmental stressors. *T. satureioides* contains a range of bioactive compounds such as thymol, carvacrol, and rosmarinic acid, which have been shown to have anti-aging, anti-inflammatory, and antimicrobial properties [6]. Many of these phytoconstituents have been incorporated into various dermacosmeceutical products such as creams, lotions, and serums. These products have been shown to improve skin hydration, elasticity, and firmness while reducing the appearance of fine lines and wrinkles [15]. Additionally, *T. satureioides* has been found to have a protective effect against UV radiation, which is a major contributor to skin damage and aging [6].

*Pseudomonas aeruginosa* is a Gram-negative, aerobic, rod-shaped bacterium that is commonly found in soil, water, and moist environments. This bacterium is known to cause a variety of infections, including skin and wound infections [16]. *P. aeruginosa* is one of the most common bacteria responsible for wound infections, particularly in immunocompromised individuals or those with chronic wounds. The skin and wound manifestations of *P. aeruginosa* infections can vary widely depending on the severity of the infection and the location of the wound. Common symptoms of *P. aeruginosa* infections include redness, swelling, pain, and pus formation. In severe cases, the infection may progress to tissue destruction, necrosis, and sepsis, which can be life-threatening [17]. Effective treatment of *P. aeruginosa* infections requires a combination of antimicrobial therapy and wound management strategies [18]. In this context, the search for natural products that could counteract the skin and wound manifestations of *P. aeruginosa* infections is an attractive strategy for healthcare professionals.

Besides their flavor and medicinal properties, *Thymus* species are also known for their mineral and nutritional minerals, such as calcium, magnesium, potassium, and iron, which are important for various functions including bone health, nerve transmission, and muscle contraction [19]. They also contain various vitamins, such as vitamins A, C, and K, which have antioxidant properties and are important for maintaining healthy skin, vision, and blood clotting [19].

The review of the existing literature has revealed that there have been no studies so far on the potential nutritional value of *T. satureioides*. Therefore, the current study aimed to examine the chemical components, nutraceutical value, and mineral contents of the plant and explore its antioxidant properties. Additionally, the extract was also evaluated against *P. aeruginosa* growth and virulence factors, including biofilm formation, protein and exopolysaccharide production, and swarming and swimming mobilities. We also performed an in silico study and discussed the potential mechanisms of action of the plant’s bioactive compounds, their potential applications in the dermatological industry, and their safety profile.

## 2. Results

### 2.1. Mineral Composition

*T. satureioides* contains high contents of calcium and iron, moderate levels of magnesium, manganese, and zinc, and low contents of total nitrogen, total phosphorus, total potassium, and copper (Table 1).

### 2.2. Amino Acid Contents

Amino acid analysis showed that asparagine, 4-hydroxyproline, isoleucine, and leucine are present in relatively high concentrations compared to the other amino acids (Table 2). Moreover, the essential amino acids represent 60.8% of the total amino acids, which is a relatively high value and indicates good protein quality.

### 2.3. LC-MS Profiling

**Figure 1:**
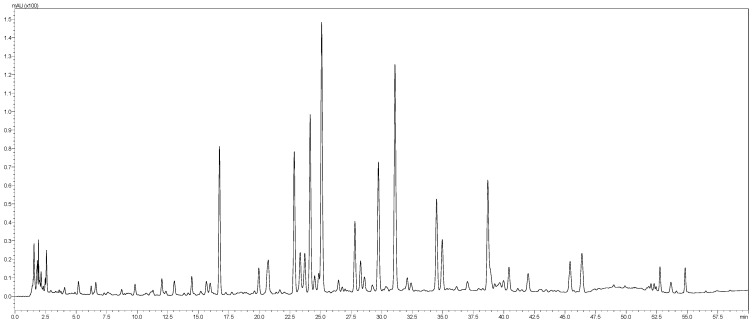
LC-MS profile of *T. satureioides* aerial parts extract.

The aqueous extract of *T. satureioides* aerial parts was analyzed by the HPLC-PDA-MS/MS. The phytoconstituents were tentatively identified using their MS, MS^2^ fragments and retention times. In total, 46 secondary metabolites belonging to phenolic acids, chalcones, and flavonoids were identified (Table 3 and Figure 1).

### 2.4. Phytocontents and In Vitro Antioxidant Assays

The extract contains substantial amounts of polyphenols and flavonoids (Table 4). Accordingly, the extract displayed noticeable antioxidant activity (Table 4). Comparatively, the standard antioxidant, quercetin, showed a significantly higher antioxidant effect, as evidenced by a lower IC_50_ in both assays.

### 2.5. Antibacterial Activities

**Figure 2:**
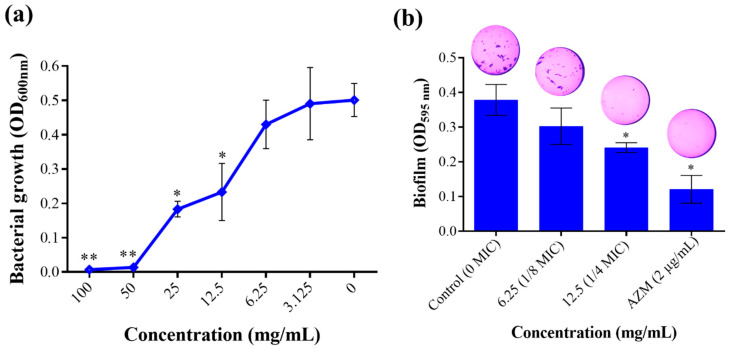
Effect of *T. satureioides* aqueous extract on (**a**) bacterial growth rate and (**b**) biofilm production by *P. aeruginosa* cultured in the absence and presence of the extract at sub-inhibitory concentrations. Asterisks (*) indicate a significant difference compared to the control media at *p* < 0.05. Asterisks (**) indicate a significant difference compared to the control media at *p* < 0.01.

In this study, *T. satureioides* aqueous extract was able to completely inhibit the growth of *P. aeruginosa* at a MIC of 50 mg/mL (Figure 2a). Noteworthy, the extract exhibited an inhibitory effect in a dose-dependent manner. Moreover, as targeting bacterial virulence factors is being explored as a novel approach to weakening microbial pathogenicity [20], we evaluated the effect of the extract on the ability of *P. aeruginosa* to form biofilm communities as well as their swimming and swarming mobilities. These two parameters are among the most important virulence determinants, allowing bacteria to form protected bacterial communities and move over biological surfaces [21,22]. Noticeably, *T. satureioides* extract at the 1/4 sub-MIC (12.5 mg/mL) substantially reduced the biofilm produced with an inhibition percentage of up to 35.13% (Figure 2b).

**Figure 3:**
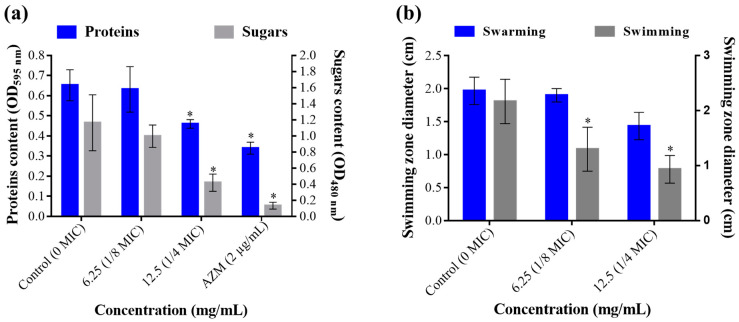
Effect of *T. satureioides* aqueous extract on (**a**) total extracellular proteins and sugar contents and (**b**) swarming and swimming mobilities of *P. aeruginosa* in the absence and presence of the extract at sub-inhibitory concentrations. Asterisks (*) indicate a significant difference compared to the control media (*p* < 0.05).

Antimicrobials are also known to interfere with many virulence factors secreted by pathogenic bacteria, including toxins, exo-proteases, adhesins, and exopolysaccharides. In this study, we demonstrated that *T. satureioides* aqueous extract at ¼ MIC (12.5 mg/mL) induced a significant reduction in the amount of extracellular proteins and sugar contents (Figure 3a). The extract showed high performance in inhibiting proteins’ secretion compared to the positive control, azithromycin (2 µg/mL), with up to 46.15% inhibition. Likewise, exopolysaccharides were significantly decreased by up to 69.04% as compared to untreated media (Figure 3a).

In addition to virulence-related molecules, bacteria are endowed with different types of mobilities, allowing them to find a host, evade host immune responses, colonize tissues, and disperse and spread [21,23]. Here, we monitored the effect of the sub-inhibitory concentrations of *T. satureioides* aqueous extract on the swimming and swarming mobility of *P. aeruginosa* on plates. This showed that the extract significantly reduced the swimming (up to 56.94% decrease) mobility of the bacterium in a dose-dependent manner (Figure 3b). In addition, even though the extract also reduced the swarming zone by up to 27.04%, ANOVA analysis showed no significant differences compared to the negative control plates (0 mg/mL) (Figure 3b).

### 2.6. Skin Permeability and Sensitization Effects of Identified Compounds

In order to computationally evaluate the skin sensitization of the identified compounds, we used the SkinSensDB database, which is based on adverse outcome pathway (AOP)-based computational prediction models. Out of the 46 identified compounds, 33 were predicted to be exempt from any skin sensitivity risk (Human Sensitizer Score ≤ 0.5) (Table 1).

The prediction of the permeability coefficient (log *K_p_*) for the transport of compounds through the mammalian epidermis is based on the linear model developed by Potts R.O. and Guy R.H. (1992) [24]. The more negative the log *K_p_* value, the less skin-permeant the molecule. Here, the log *K_p_* values were computed using the SwissADME online tool. The 46 compounds showed extensive skin permeability abilities, with log *K_p_* values ranging from −3.35 cm/s (Phloretic acid caffeate) to −10.90 cm/s (Eriodictyol rutinoside) (Table 1). For instance, dimethyl quercetin and apigenin were shown to have higher skin permeability (−5.99 and −5.80 cm/s) than quercetin pentoside and galloyl glucose (−10.54 and −10.66 cm/s, respectively).

## 3. Discussion

*T. satureioides* is a species of thyme that is widely distributed in the Mediterranean region. This herb has been traditionally used as a medicinal plant, and recent studies have demonstrated its potential for a wide range of health benefits. In this study, we showed that the aqueous extract of its aerial parts contains a variety of phytochemicals. We also demonstrated that it is endowed with strong antioxidant, antibacterial, antibiofilm, and anti-quorum sensing activities in vitro.

After reviewing subsequent and more recent literature, we noticed that the nutritional value of *T. satureioides* has never been examined so far. Therefore, we analyzed the mineral content of *T. satureioides*. Although the normal mineral contents in medicinal plants can vary greatly depending on the specific plant species, its growing conditions, and the part of the plant being studied, general ranges showed that *T. satureioides* contains high contents of calcium and iron, moderate levels of magnesium, manganese, and zinc, and low contents of total nitrogen, total phosphorus, total potassium, and copper. This suggests that the plant may be a good source of calcium and iron, as well as other important minerals. Comparatively to closely related *thymus* species such as *T. vulgaris*, *T. satureioides* contains higher levels of iron (591.25 versus 174.5 mg/Kg DW) and manganese (48.6 versus 17.19 mg/Kg DW) but lower contents of zinc (14.66 versus 18.1 mg/Kg DW) [19]. Noteworthy, *T. vulgaris* was also shown to be rich in some important vitamins such as folic acid, B complex, β-carotene, and vitamins A, K, E, and C [19]. Hence, determining the vitamin content of *T. satureioides* would be of interest to extend the health benefits of this species.

In addition to the mineral content analysis, we also profiled the phytoconstituents of the extract using LC/MS-MS. We detected 46 compounds belonging mainly to phenolic acids, chalcones, and flavonoids. These include apigenin, quercetin, luteolin, and rosmarinic and salvianolic acids. Previous studies corroborate this phytochemical profile, where some similar compounds were identified from *T. satureioides,* mainly apigenin, luteolin, chlorogenic acid, feruloyl caffeic acid, and rosmarinic acid, among others [6]. The richness of this species in phenolic compounds and flavonoids is most likely responsible for its strong antioxidant properties. These compounds act as free radical scavengers and prevent cellular damage caused by oxidative stress, thus maintaining overall health and preventing chronic diseases such as cancer, diabetes, cardiovascular diseases, and age-related damage [25]. Here, we showed that the extract contains a high content of total phenolics and flavonoids and exhibits a strong antioxidant potential using both DPPH and ABTS assays. Similar observations were reported showing that *T. satureioides* extracts were the richest in total phenolics (up to 70.2 mg GAE/g) and flavonoids (up to 52.7 mg QuE/g) compared to the other extracts from *Petroselinum crispum* and the microalgae *Spirulina platensis* [26]. Elsewhere, the aqueous extract of *T. satureioides* was also shown to elicit ferric-reducing antioxidant power (FRAP) at up to 7.03 ± 0.29 mmol trolox/g [27]. Interestingly, salvianolic acid A, a compound detected in the tested extract, was previously shown to protect retinal pigment epithelial cells against oxidative stress via activating Nrf2/HO-1 signaling pathways [28]. In addition to these compounds, *T. satureioides* is also known to contain high amounts of essential oils, which contribute to its aromatic and flavor properties [29]. 

Although there is substantial data in the literature justifying the traditional use of *T. satureioides* in skin diseases [6,30,31], there is no biological and/or pharmacological experimental evidence regarding these claims. In this regard, the dermacosmeceutical potential of *T. satureioides* is one of the main original aspects addressed in this study. It was investigated by assessing the extract’s effect against *P. aeruginosa*, a main pathogenic bacterium with common skin-related symptoms. These can include redness, inflammation, and the formation of pus-filled lesions known as pyoderma. *P. aeruginosa* infections can also cause a condition called hot tub folliculitis, which is characterized by a rash of small, red, itchy bumps that appear after exposure to contaminated water sources, such as hot tubs. In severe cases, *P. aeruginosa* infections can lead to tissue necrosis, which can cause extensive tissue damage. The skin manifestation of *P. aeruginosa* infections can be particularly problematic for immunoexpressed subjects [18,32,33]. The antibacterial effect of *T. satureioides* was largely investigated using its essential oil against a large spectrum of species, including *Enterobacter cloacae*, *Staphylococcus aureus*, *Acinetobacter baumannii*, *Escherichia coli,* and *Bacillus cereus* [34]. In this work, we tested the aqueous extract of *T. satureioides* aerial parts and showed that it has a strong antibacterial effect against *P. aeruginosa*. In addition, lower concentrations, sub-MICs (6.25 and 12.5 mg/mL), were potent and significantly reduced the biofilm formation abilities of the bacterium. Biofilm formation by *P. aeruginosa* is a critical aspect of its pathogenicity, as it allows the bacterium to colonize and persist in a wide range of environments, including medical devices and human tissues. They also make the bacterium more resistant to many antibiotics [35].

Compared to other thymus species, Noumi et al. (2023) showed that the methanolic extract of *Thymus musilii* Velen. was able to completely inhibit the growth of *P. aeruginosa* at MIC = 12.5 mg/mL and induced high inhibitory effects on its ability to swarm (up to 39.73 ± 1.50%) and swim (up to 25.18 ± 1.00%). Noticeably, *T. musilii* extract did not significantly inhibit the biofilm formation at >12.5 mg/mL for *P. aeruginosa,* while it impaired its formation by *Salmonella typhimurium* (53.96 ± 4.21%) and *Listeria monocytogenes* (49.54 ± 4.5%) [36]. Therefore, *T. satureioides* and its phytoconstituents could be promising in counteracting the ability of *P. aeruginosa* to form biofilms during infections by inhibiting the formation of a protective matrix of bacterial communities and thus their adherence to biological surfaces. In addition to biofilm formation, *P. aeruginosa* produces a variety of virulence factors, including proteases, that contribute to its ability to cause infections in humans. The combination of biofilm formation and virulence factor production makes *P. aeruginosa* a serious pathogen that poses a significant threat to public health. In this study, total extracellular protein quantification revealed that *T. satureioides* extract, at the sub-MIC of 12.5 mg/mL, was able to significantly reduce protein secretion by the bacterium by up to 46.15%. Similarly, secreted exopolysaccharides were significantly decreased by up to 69.04% when culture media contained 12.5 mg/mL of the extract. These anti-quorum sensing effects were also investigated using *Mangifera indica* L. extract at 800 µg/mL, which was demonstrated to be potent in reducing total protease (56%), elastase (76%), chitinase (55%), exopolysaccharide secretion (58%), and swarming motility (74%), in *P. aeruginosa* PAO1 [37]. Our findings demonstrated that *T. satureioides* phytocompounds could target bacterial genes involved in proteases (e.g., lasR, lasI, rhlI, rhlR, exoU, lasB) and biofilm (e.g., pslA, pelA, ppyR) synthesis [38]. In fact, quercetin, one of the major compounds identified in our plant extract, was previously shown to be effective in reducing the expression of several quorum sensing genes, lasR, lasI, rhlI, and rhlR, by 68, 34, 57, and 50%, respectively [39]. Indeed, many studies have corroborated the potency of plant-based extracts and their phytoconstituents to impair bacterial virulence factors. For instance, the aqueous extracts from *Conocarpus erectus*, *Callistemon viminalis*, and *Bucida buceras* were investigated for their impact on *P. aeruginosa*’s virulence factors and quorum sensing. The findings showed that all three plants significantly suppressed LasA protease, LasB elastase, pyoverdin production, and biofilm formation. Moreover, each plant had a different effect on the Las and Rhl genes and their corresponding signaling molecules, highlighting that different mechanisms were involved in their effectiveness [40]. Outstandingly, the effect of *T. satureioides*, especially its polar extracts, has not been examined for their anti-quorum sensing activities. Considering these preliminary data, the utility of the anti-quorum sensing properties of *T. satureioides* extract in combating *P. aeruginosa* infections by preventing the formation of biofilms, reducing the production of toxins, and impairing the ability of bacteria to colonize and infect host tissues seems attractive. This approach may have several advantages over traditional antibiotics, such as reducing the risk of antibiotic resistance and promoting wound healing [41].

Besides phytochemicals, minerals, and vitamins, amino acids are very important in the dermacosmeceutical industry as they contribute to triggering different signaling pathways within human skin cells, which activate particular genes associated with aging and safeguard the cells from stress factors [42,43]. Interestingly, it was suggested that hydrophobic amino acids significantly contribute to the antioxidant ability of natural products [44]. In the present study, we noticed that *T. satureioides* is rich in asparagine, 4-hydroxyproline, isoleucine, and leucine compared to the other amino acids characterized, indicating that they may be important for protein function and structure in the plant. The presence of 4-hydroxyproline is noteworthy as it is a non-standard amino acid that is usually found in collagen, the main structural protein in animals, and it is less common in plant proteins. In addition, 4-hydroxyproline is a critical element in stabilizing the triple helical conformation of collagen [45], a protein that conforms the supportive and connecting tissues in the skin [46]. Moreover, the ratio of essential amino acids (isoleucine, leucine, phenylalanine, threonine, valine, histidine, and lysine) to non-essential amino acids (alanine, aspartic acid, asparagine, glutamic acid, serine, and tyrosine) can reflect the protein quality of the plant. Noticeably, the essential amino acids represented 60.8% of the total amino acids, which is a relatively high value and indicates good protein quality. The amino acid composition of *T. satureioides* suggests that it may have a nutritionally valuable protein content and could be integrated as an active ingredient in formulating dermacosmeceutical agents [44]. 

An important advancement in the field of toxicology in recent times has been the utilization of alternative methods to animal testing. These methods, including those for skin sensitization, have gradually been integrated into regulatory practices [47]. Hence, as topical dermacosmeceuticals are a popular category of skincare products that are formulated with active ingredients, ensuring the safety of consumers is mandatory and constitutes a critical concern in their manufacturing. Indeed, skin sensitization is a crucial aspect of the process of developing drugs and making regulatory decisions. Chemical sensitizers, including plants, act by binding to proteins, causing immune responses that may lead to allergic contact dermatitis [48]. Sensitization is a process where the body’s immune system reacts to a substance that it recognizes as foreign, even though the substance may not be harmful [49]. Here, out of the 46 identified compounds, 33 were predicted to be exempt from any skin sensitivity risk (Human Sensitizer Score ≤ 0.5). In addition, the compounds showed diverse skin permeabilities, with log *K_p_* values ranging from −3.35 cm/s (Phloretic acid caffeate) to −10.90 cm/s (Eriodictyol rutinoside). This shows that most of the plant phytochemicals are potentially safe agents for dermatological applications. Nevertheless, dermatotoxicity assays in vivo involving skin irritation (e.g., occluded dermal irritation test method), skin sensitization (e.g., Mouse Ear Swelling Test (MEST)), acute dermal toxicity, and repeated toxicity tests would be mandatory to bridge the knowledge gap on the cosmeceutical and dermatological applications of *T. satureioides*.

## 4. Material and Methods

### 4.1. Plant Material and Extract Preparation 

Flowering aerial parts of *T. satureioides* Coss. were purchased from a local herbalist in Ben Guerir, Morocco. The dried plant material was subjected to hydro-distillation (150 g/L, *w*/*v*) utilizing a Clevenger apparatus until no more essential oil (EO) was found. The residual water from the hydro-distillation process was collected, filtered, evaporated under reduced pressure (BUCHI, Flawil, Switzerland), and lyophilized to obtain the crude extract (24.7 g).

### 4.2. Mineral Analysis

Nitrogen content was analyzed using the Kjeldahl method [50], while inductively coupled plasma optical emission spectrometry (ICP-OES) was utilized to estimate the levels of total phosphorus, potassium, calcium, magnesium, sodium, iron, manganese, zinc, and copper. 

### 4.3. LC-MS Analysis

The phytochemical characterization of the extract was performed using the HPLC-PDA-MS/MS system consisting of a Shimadzu Japan system (Tokyo, Japan) coupled to an MS 8050 mass spectrometer with an electrospray ionization (ESI) source, as previously described [51]. 

### 4.4. Analysis of Amino Acids 

Amino acid characterization and quantification were analyzed using high-performance liquid chromatography (HPLC) tandem mass spectrometry (Shimadzu 8050, Tokyo, Japan) [51]. Forty mg of the plant extract was hydrolyzed in 10 mL of 6 M HCl for 22 h at 110 °C. The hydrolyzed sample was cooled down to 4 °C to stop the hydrolyzation process and then diluted in 50 mL of distilled water. The hydrolysate pH was adjusted to 4.5 and filtered using a 0.22-micrometer PTFE membrane to remove suspended particles. Liquid chromatography was performed at 40 °C using a Shim-pack GIST PFPP Kyoto, Japan (2.1 mm I.D. × 150 mm, 3.0 mm) column and a gradient system with the mobile phase consisting of solvent water and acetonitrile with 0.1% of formic acid in each solvent, at a flow rate of 0.25 mL/min, and an injection volume of 3 μL. The gradient program used 0–2 min 100% of A, 5 min 75% of A, 11 min 65% of A, 16 min 50%, 19 min 5%, 30 min 100% of B for 2 min, and hold for 4 min. The conditions of mass spectroscopy were in ESI positive and negative modes.

### 4.5. Phytocontents

The total polyphenolic content (TPC) in a 96-well microplate was determined by utilizing the Folin-Ciocalteu method [52]. Firstly, 20 μL of the extract with a concentration of 1 mg/mL was added to the microplate, followed by the addition of 100 μL of Folin-Ciocalteu reagent (10% *v*/*v*). The mixture was then incubated for 5 min in the dark at room temperature, after which 80 μL of 7.5% Na_2_CO_3_ (*w*/*v*) was added and mixed thoroughly. The resulting mixture was then incubated for an additional 30 min in the dark at room temperature, and its absorbance was measured at 765 nm using a UV-vis spectrophotometer (BMG LABTECH, Bath, UK). The TPC was expressed as mg GAE/g DW (micrograms of gallic acid equivalents per milligram of dry weight extract), and a calibration curve was established using gallic acid as the standard. The experiment was carried out in triplicate for all samples.

The total flavonoid content (TFC) in the extract was detected using the aluminum chloride (AlCl_3_) method [53]. To prepare the reaction mixture, 100 μL of the extract with a concentration of 1 mg/mL was combined with 0.5 μL of AlCl_3_ (1.2%) and 0.5 μL of potassium acetate (120 mM). The mixture was allowed to stand for 30 min at room temperature, and the absorbance of the reaction mixture was measured at 415 nm. The TFC was expressed as μg of quercetin equivalent per mg of dry weight extract (μg QE/mg DW), and all samples were tested in triplicate.

### 4.6. Antioxidant Activity In Vitro

#### 4.6.1. DPPH Radical Scavenging Assay

The extract’s ability to scavenge DPPH was assessed using a modified spectrophotometric method [54]. Briefly, 100 μL of the extract and 100 μL of a 0.2 mM DPPH solution were combined in each well of a 96-well plate. The plate was then kept in the dark at room temperature for 30 min, after which the absorbance was measured at 517 nm. Quercetin was employed as a standard (1 to 1000 μg/mL), and all samples were tested in triplicate. The scavenging capacity was determined using the following equation:

$$Scavenging\;effect\left(\%\right)=\left[\frac{Abs\;control-Abs\;sample}{Abs\;control}\right]\times 100$$

#### 4.6.2. ABTS Radical Scavenging Assay

The ABTS radical cation method was modified to evaluate the free radical-scavenging effect of the extract [55]. First, ABTS^+^ was generated by oxidizing ABTS with potassium persulfate. The resulting mixture was then kept in the dark at room temperature for 16 h. Afterward, the mixture was diluted with ethanol to an absorbance of 0.7 ± 0.02. Next, 180 μL of the ABTS reagent was mixed with 20 μL of the extract in a 96-well microplate, and the mixture was incubated at room temperature for 6 min. After the incubation period, the absorbance was measured at 734 nm. All determinations were carried out in triplicate. The ABTS scavenging effect was calculated using the following formula:

$$Radical\;scavenging\left(\%\right)=\left[\frac{Abs\;control-Abs\;sample}{Abs\;control}\right]\times 100$$

The IC_50_ ABTS values were obtained through extrapolation from the regression analysis.

### 4.7. Antibacterial Activity

#### 4.7.1. Minimal Inhibitory Concentration (MIC) Determination

The antibacterial activity of *T. satureioides* extract was tested using the microdilution assay in a sterile 96-well microplate [56,57]. The extract was solubilized in Mueller Hinton (MH) broth, filtered using 0.22 μm sterile syringe filters, two-fold serially diluted (100, 50, 25, 12.5, and 6.25 mg/mL), and then transferred into the microplate’s wells in triplicates (200 μL per well). Afterward, 5 μL of a fresh overnight culture of *P. aeruginosa* adjusted to an OD_600nm_ of 0.6 were inoculated into each well and incubated at 37 °C under 150 rpm shaking for 18 h. The minimum inhibitory concentration (MIC) corresponds to the lowest concentration that inhibits visible microbial growth. The bacterial viability was checked at OD_600nm_. The extract-free media as well as the uninoculated MH media were used as controls.

#### 4.7.2. Bacterial Biofilm Inhibition Assay

The anti-biofilm effect of the extract was evaluated using the crystal violet colorimetric assay [58,59,60]. Firstly, the plant extract at 6.25 mg/mL (1/8 MIC) and 12.5 mg/mL (1/4 MIC) was filtered using 0.22 μm sterile syringe filters, inoculated, and incubated as described above. Media without bacterial inoculation were used as negative controls. After 18 h of incubation, the culture suspensions were discarded, and the wells were washed four times with a PSB solution to eliminate planktonic bacteria. Next, each well was filled with 1% crystal violet and kept for 15 min at room temperature. Afterward, a vigorous washing with distilled water was carried out to remove any excess dye. The biofilm was solubilized by 95% ethanol and quantified at OD_595nm_ using a multimode plate reader. Azithromycin at 2 µg/mL was used as the standard antibiotic [37]. 

#### 4.7.3. Swimming and Swarming Inhibition Assays

*T. satureioides* extract was also evaluated for its potential effect on the swimming and swarming motilities of *P. aeruginosa*. The concentrations of 6.25 mg/mL (1/8 MIC) and 12.5 mg/mL (1/4 MIC) of the extract were aseptically added to the swimming (1% tryptone, 0.5% sodium chloride, and 0.3% agar) and swarming (semisolid LB medium, 0.6%) media [61]. Next, 10 μL of a fresh overnight culture of *P. aeruginosa* (OD_600nm_ = 1) was deposited at the center of each plate and incubated at 37 °C for 18 h. The mobilities’ zone diameters were measured in cm [56,58]. Extract-free media were used as the negative control.

#### 4.7.4. Outer Membrane Protein Quantification 

To evaluate the effect of the extract on the amount of virulence factors produced by the bacterium, the outer membrane proteins were quantified using the Bradford assay in a sterile 96-well microplate [62,63]. Briefly, *P. aeruginosa* was cultivated in the presence (1/8 and 1/4 MIC) and absence of the extract for 24 h. The cultures were centrifuged (10,000× *g*; 12 min), and the supernatants were filtered using 0.45 μm syringe filters. The filtered supernatants were used to quantify the total protein content at OD 595 nm. Azithromycin at 2 µg/mL was used as the standard molecule, and uninoculated media were used as the blank controls [37].

#### 4.7.5. Exopolysaccharide Estimation

In addition to the total protein content, the supernatants were used to extract the EPS by the addition of chilled ethanol (95%) to completely precipitate the EPS and left overnight to precipitate the EPS at 4 °C. The concentrations of sugar content were determined using the protocol of [37,64]. Azithromycin at 2 µg/mL was used as the standard molecule, and media without inoculum were used as blanks [37].

### 4.8. In Silico Suitability for Skin Application

The rate at which a phytocompound can penetrate the stratum corneum, known as skin permeability (log *K_p_*), is a commonly used parameter to quantify the transport of molecules across the outermost layer of the epidermis and indicate the extent of skin absorption. We checked all identified compounds for log *K_p_* using the SwissADME database [65]. Furthermore, we assessed the skin sensitization potential of identified compounds using the human skin sensitizer score from the SkinSens database (available at: https://cwtung.kmu.edu.tw/skinsensdb/, accessed on 1 June 2023). The score is based on a scale from 0 to 1, with phytochemicals that score 0 being the least skin-sensitizing and those that score 1 being the most skin-sensitizing. Compounds scoring above a certain threshold (≥0.5) are considered to be skin sensitizers.

### 4.9. Statistical Analysis

Data analysis was performed in IBM SPSS Statistics 20 software using one-way analysis of variance (ANOVA) followed by many-to-one comparisons according to the post-hoc analysis with Dunnett’s test. The data represent the mean and standard deviation of three independent experiments. Each treatment was compared to a single control. Significant differences were set at *p* < 0.05.

## 5. Conclusions

In the present study, we demonstrated that *T. satureioides* is endowed with a wide range of nutritional, dermatological, and anti-quorum sensing properties. The plant contained high levels of calcium and iron, moderate amounts of magnesium, manganese, and zinc, and low levels of nitrogen, phosphorus, potassium, and copper. It is rich in amino acids, with essential amino acids comprising a substantial portion. The extract exhibited potent antioxidant activity, inhibited the growth of *P. aeruginosa*, reduced biofilm formation, and impacted bacterial swimming and extracellular protein and exopolysaccharide production. The plant’s compounds were also examined for their suitability for dermatological applications via in silico toxicity assessment of the skin sensitivity score and permeability index. This revealed a low skin sensitivity risk for most identified compounds and highlighted varying degrees of skin permeability. This makes the plant and its phytochemicals promising candidates for use in the development of new drugs and food supplements. Nevertheless, further research is needed to explore the full potential of *T. satureioides*, understand its mechanisms of action and potential dermatotoxicities, and develop effective formulations for various skin disorders.

## Figures and Tables

**Table 1:** Mineral composition of *T. satureioides* aqueous extract.

Parameter	Unit	Result	Method Used
Total nitrogen	% DW	0.98	Kjeldahl
Total phosphorus		0.09	ICP-OES
Total potassium		0.95	
Calcium		1.13	
Magnesium		0.18	
Sodium	mg/Kg DW	85.74	
Iron		591.25	
Manganese		48.6	
Zinc		14.66	
Copper		6.49	

DW: dry weight; ICP-OES: inductively coupled plasma-optical emission spectrometry.

**Table 2:** Amino acid composition of *T. satureioides*.

Analyte	Concentration (mg/kg)
Aspartic acid	0.064
Serine	0.039
Asparagine	5.58
4-Hydroxyproline	13.19
Glutamic acid	0.21
Threonine	0.17
Lysine	0.08
Alanine	0.68
Valine	0.11
Histidine	0.32
Tyrosine	4.72
Leucine	14.75
Isoleucine	15.95
Phenylalanine	5.66

**Table 3:** Polyphenolics from *T. satureioides* aerial parts extract.

No.	Rt (min)	[M − H]^−^	MS/MS	Proposed Secondary Metabolites	Log *K_p_* (cm/s) *	Human Sensitizer (Score) ^¥^
1	1.46	191	127	Quinic acid	−9.15	0.635
2	1.63	133	115	Malic acid	−8.01	0.200
3	3.68	331	169	Galloyl glucose	−10.66	0.570
4	4.88	315	153	Dihydroxybenzoic acid glucoside	−9.12	0.430
5	6.20	153	109	Dihydroxybenzoic acid	−6.39	0.425
6	7.22	353	191	Chlorogenic acid	−8.76	0.565
7	7.82	299	137	Hydroxybenzoic acid glucoside	−8.73	0.290
8	8.60	339	177	Aesculetin glucoside	−8.81	0.300
9	9.27	341	179	Caffeoyl glucose	−9.94	0.495
10	11.07	353	191	Neochlorogenic acid	−8.76	0.565
11	13.05	305	225	Gallocatechin	−8.17	0.545
12	14.44	325	163	Coumaroyl glucose	−8.34	0.460
13	15.40	337	191	Coumaroylquinic acid	−8.41	0.560
14	15.52	307	227	Resveratrol sulphate	−6.38	0.765
15	15.88	327	165	Phloretic acid caffeate	−3.35	0.360
16	16.60	593	353	Apigenin di-*C*-glucoside	−11.53	0.270
17	19.79	431	269	Apigenin glucoside	−8.07	0.315
18	20.33	593	353	Apigenin di-*C*-glucoside	−11.53	0.270
19	20.57	477	301	Quercetin glucuronide	−8.78	0.520
20	20.81	609	301	Quercetin rutinoside	−10.26	0.400
21	21.29	593	353	Apigenin di-*C*-glucoside	−11.53	0.270
22	21.89	623	285	Luteolin glucosyl glucuronide	−8.00	0.600
23	22.91	595	287	Eriodictyol rutinoside	−10.90	0.375
24	23.99	593	285	Luteolin rutinoside	−10.06	0.305
25	24.65	447	285	Luteolin glucoside	−8.00	0.265
26	25.20	461	285	Luteolin glucuronide	−8.25	0.600
27	25.32	609	315	Isorhamnetin pentosyl glucoside	−8.73	0.320
28	26.16	521	359	Caffeoyl rosmarinic acid	−6.82	0.480
29	27.67	577	269	Apigenin rutinoside	−11.98	0.295
30	28.60	755	285	Luteolin caffeoyl rutinoside	−10.06	0.305
31	28.75	433	301	Quercetin pentoside	−10.54	0.435
32	28.81	445	269	Apigenin glucuronide	−7.99	0.520
33	28.99	355	179	Feruloyl caffeic acid	−6.58	0.320
34	29.16	431	269	Apigenin glucoside	−8.07	0.315
35	29.58	609	301	Quercetin coumaroyl glucoside	−8.14	0.375
36	30.61	515	353	Dicaffeoylquinic acid	−8.37	0.605
37	30.79	475	299	Diosmetin glucuronide	−7.76	0.385
38	31.21	359	197	Rosmarinic acid	−6.82	0.480
39	32.29	549	387	Caffeoyl ethyl rosmarinate	−6.82	0.480
40	32.47	551	197	Schizotenuin F	−6.81	0.380
41	34.33	493	197	Salvianolic acid A	−6.53	0.485
42	34.52	537	197	Salvianolic acid I	−6.96	0.625
43	38.60	493	197	Salvianolic acid A isomer		
44	39.26	537	197	Salvianolic acid I isomer		
45	41.83	329	271	Dimethyl quercetin	−5.99	0.490
46	45.52	269	151	Apigenin	−5.80	0.625

* The skin permeation coefficient. ^¥^ The skin sensitization potential of identified compounds using the human skin sensitizer score from the SkinSens database.

**Table 4:** Phytocontents and in vitro antioxidant activities of the aqueous extract of *T. satureioides* aerial parts.

Parameter	*T. satureioides* Aqueous Extract	Quercetin
TPC (mg GAE/g extract)	118.17 ± 2.25	-
TFC (mg quercetin/1 g extract)	32.32 ± 1.07	-
DPPH (IC_50_ μg/mL)	22.28 ± 2.34 *	2.20 ± 0.15
ABTS (IC_50_ (μg/mL)	157.08 ± 5.37 *	17.26 ± 1.87

Asterisks (*) indicate a significant difference compared to the reference compound (*p* < 0.05). GAE: gallic acid; TPC: total polyphenol content; TFC: total flavonoid content.

## Data Availability

The data presented in this study is contained within the article.
